# Adam33 polymorphisms are associated with COPD and lung function in long-term tobacco smokers

**DOI:** 10.1186/1465-9921-10-21

**Published:** 2009-03-12

**Authors:** Alireza Sadeghnejad, Jill A Ohar, Siqun L Zheng, David A Sterling, Gregory A Hawkins, Deborah A Meyers, Eugene R Bleecker

**Affiliations:** 1 Center for Human Genomics and Department of Medicine and Pediatrics, Wake Forest University School of Medicine, Winston-Salem, North Carolina, USA; 2 School of Public Health, Saint Louis University, St. Louis, Missouri, USA

## Abstract

**Background:**

Variation in ADAM33 has been shown to be important in the development of asthma and altered lung function. This relationship however, has not been investigated in the population susceptible to COPD; long term tobacco smokers. We evaluated the association between polymorphisms in ADAM33 gene with COPD and lung function in long term tobacco smokers.

**Methods:**

Caucasian subjects, at least 50 year old, who smoked ≥ 20 pack-years (n = 880) were genotyped for 25 single nucleotide polymorphisms (SNPs) in ADAM33. COPD was defined as an FEV1/FVC ratio < 70% and percent-predicted (pp)FEV1 < 75% (n = 287). The control group had an FEV1/FVC ratio ≥ 70% and ppFEV_1 _≥ 80% (n = 311) despite ≥ 20 pack years of smoking. Logistic and linear regressions were used for the analysis. Age, sex, and smoking status were considered as potential confounders.

**Results:**

Five SNPs in ADAM33 were associated with COPD (Q-1, intronic: p < 0.003; S1, Ile → Val: p < 0.003; S2, Gly → Gly: p < 0.04; V-1 intronic: p < 0.002; V4, in 3' untranslated region: p < 0.007). Q-1, S1 and V-1 were also associated with ppFEV1, FEV1/FVC ratio and ppFEF25–75 (p values 0.001 – 0.02). S2 was associated with FEV1/FVC ratio (p < 0.05). The association between S1 and residual volume revealed a trend toward significance (p value < 0.07). Linkage disequilibrium and haplotype analyses suggested that S1 had the strongest degree of association with COPD and pulmonary function abnormalities.

**Conclusion:**

Five SNPs in ADAM33 were associated with COPD and lung function in long-term smokers. Functional studies will be needed to evaluate the biologic significance of these polymorphisms in the pathogenesis of COPD.

## Background

Chronic Obstructive Pulmonary Disease (COPD) is a disorder that is characterized by progressive decline in lung function. The rate of decline in FEV1 in long term tobacco smokers who are susceptible to tobacco smoke is 3–5 fold that of the normal age related decline [1,2]. Nearly 90% of COPD is caused by long term cigarette smoking; however, only 25% of chronic tobacco smokers develop COPD [3]. Tobacco exposure in pack years correlates weakly with FEV1 [4] however, this relationship only partially explains reduced lung function in cigarette smokers with COPD. Furthermore, hyperinflation indicated by an enlarged residual volume is present in a subset of individuals with COPD while others manifest primarily a chronic bronchitic phenotype. Thus, host or genetic factors appear to predispose some individuals with tobacco exposure to the development of smoking related respiratory disease. Additionally, COPD tends to occur more frequently in smokers with a family history of obstructive airways disorders such as asthma and COPD. Thus, it has been suggested that asthma and COPD may share some predisposing factors and some clinical characteristics (The Dutch hypothesis [5-7]).

In 2002, van Eerdewegh and coworkers identified ADAM33 as a susceptibility gene for asthma and bronchial hyperresponsivess on chromosome 20 p using positional cloning techniques [8]. While a number of studies have replicated this finding showing that ADAM33 is a susceptibility gene for asthma in different populations [9-12], some studies have not replicated these findings [13,14]. In addition variation in this gene was shown to be associated with an accelerated rate of decline in FEV1 in a longitudinal study of subjects with a clinical diagnosis of asthma [15] and with reduced lung function in a prospective birth cohort study [16]. In a longitudinal study from a general population, van Diemen and coworkers showed associations between SNPs in ADAM33 and annual decline in FEV1 in cigarette smokers who were compared to the larger population[17]. These studies did not comprehensively investigate the genetic variations observed in the ADAM33 gene and were not performed in a population of chronic cigarette smoker, the appropriate target population for studies of genetic susceptibility in COPD. Therefore, we comprehensively assessed ADAM33 variation (25 SNPs) in a large well characterized population of long term tobacco smokers and investigated the associations between these variations and COPD and spirometric variables.

## Methods

### Population and data

Subjects were recruited from a cohort of tradesmen referred for a work-related independent medical evaluation [18]. Referrals were come from trade unions as well as television and newspaper advertisements. Participants gave informed consent for their involvement in the genetic study, and the research protocol was reviewed and approved by the institutional review boards at Wake Forest University and Saint Louis University. As part of the referral process, an extensive questionnaire, a chest radiograph, and pulmonary function testing were obtained.

The questionnaire (additional file 1) detailed information about prior employment, smoking history, and personal and family medical histories. The questionnaire was self-administered prior to evaluation, and the physician examiner reviewed the entire questionnaire at the time of examination. Subjects were asked to quantify their cigarette smoking as packs per day, and ages of initiation and cessation of tobacco use. Chest radiographs were obtained and interpreted by a certified B-reader. Chest radiograph abnormalities were quantified according to the International Labor Organization (ILO) scoring system [19]. Lung function was measured at a variety of accredited hospital pulmonary function laboratories using equipment available at those sites. Pulmonary function testing was performed according to American Thoracic Society published guidelines [20]. Residual volume (RV) using He dilution was measured in a subset of subjects (FVC ≤ 80% predicted) to confirm the presence of restriction or hyperinflation [21]. Prebronchodilator spirometric data was used in the analysis.

For the current study, subjects over 50 years of age with a greater than or equal to 20 pack-year history of cigarette smoking were included in the analysis. We did not genotype any subject who was not a smoker or smoked less than 20 pack-years. The presence of evident occupational exposure induced lung disease (ILO scores greater than 1/1, 89 subjects), mesothelioma, and an anticipated survival of less than one year secondary to active cancer, or other chronic diseases (226 subjects) were exclusion criteria.

### COPD phenotype

The COPD phenotype, is a composite variable based on the GOLD guidelines [21]. However, to avoid a possible misclassification in the analyses, we classified COPD cases by using more stringent criteria. COPD was defined as an FEV1/FVC ratio < 70% and percent-predicted (pp)FEV_1 _< 75% (GOLD guideline criteria for stage 2 and above: FEV1/FVC ratio < 70% and ppFEV_1 _< 80%). Controls had an FEV1/FVC ratio ≥ 70% and ppFEV_1 _≥ 80%. Subjects who fell into the category with FEV1/FVC ratio ≥ 70% and ppFEV_1 _< 80%, or FEV1/FVC ratio < 70% and ppFEV_1 _≥ 75%, unclassified smokers, were excluded from categorical analyses (COPD vs. unaffected smokers) but included in additional analyses of continuous variables (quantitative traits: ppFEV_1_, FVC, FEV1/FVC ratio and ppFEF25–75).

### Genotyping method

To further characterize the ADAM33 gene, we genotyped the target population for 25 SNPs in the gene chosen based on the Hapmap data and supplemented by SNPs reported in previous studies (25 SNPs). SNP genotyping was performed using the MassARRAY SNP genotyping system (Sequenom, Inc., San Diego CA) which utilizes a primer extension assay followed by mass spectrometry for oligonucleotide size determination. PCR and extension primers were designed using SpectroDesigner software (Sequenom, Inc.) and reactions were performed according to the manufacturer's instructions. Genotypes were scored automatically using the SpectroTyper software (Sequenom, Inc.), and checked with quality control samples (i.e., duplicate DNA samples, negative controls) manually. All polymorphisms were assessed to determine if the observed genotype frequencies were consistent with Hardy-Weinberg equilibrium using Chi-square tests. Pair-wise marker-linkage disequilibrium was estimated using Lewontin's D' statistic and r^2 ^[22].

### Data analysis

We included 19 SNPs in ADAM33 that had a MAF ≥ 0.05. The data analysis was performed in two stages. In the first stage we evaluated the association between the SNPs and COPD assuming an additive genetic model. In the next step we explored their relationship between the SNPs that reached a nominal statistical significance (p value < 0.05) in the first step, with pulmonary function measurements. We combined minor allele homozygotes with heterozygotes at this step as they were either absent or had very low frequencies. As we considered the second step in the analysis to be exploratory and because of the fact that COPD and pulmonary function measurements are highly correlated we corrected for multiple comparison testing based on our analysis in the first step. Therefore the Bonferroni corrected p-value was calculated as 0.05/19 (0.0026).

The association between ADAM33 genotypes and COPD having unaffected smoking as controls was evaluated by Logistic regression. We controlled for sex, age and pack-years smoked. To test for association we used Chi-square test for trend, assuming that the risk of the heterozygote genotype is between the risks of the major and the minor homozygote genotypes: additive genetic model. Generalized linear models (linear regression), adjusted for sex, age and pack-years smoked were used to assess the associations between SNPs and the pulmonary function measurements: pp (percent predicted) FEV_1_, ppFVC, FEV1/FVC ratio, ppFEF_25–75 _and percent predicted residual volume (ppRV). In the quantitative trait analyses for each SNP, we combined the heterozygote genotype with the minor homozygote genotype as they showed a similar effect in primary analysis. Statistical analysis was performed using SAS software (SAS Institute, Cary, N.C.).

Haplotype analysis for the SNPs genotyped was performed using a 3 SNP sliding window approach. Tests for association between haplotypes and COPD were performed using a score test as implemented in the computer program HAPLO.SCORE [23].

## Results

Of the 880 subjects genotyped 97% of the subjects were men. Of these, 281 fell into the group excluded from categorical analyses (FEV1/FVC ratio ≥ 70% and ppFEV_1 _< 80% or FEV1/FVC ratio < 70% and ppFEV_1 _≥ 75%). The clinical characteristics of the groups with COPD, unaffected smoking controls and the unclassified cigarette smokers are shown in Table 1. They differed by FEV_1_, FEV1/FVC ratio and ppFEV_1 _because of the phenotype definition. Subjects with COPD were slightly older (67.3 vs. 64.4) and smoked 58.6 pack years compared with 45.9 pack years in unaffected smokers (Table 1). Smoking history in pack years correlated significantly (p < 0.0001) with ppFEV_1_.

**Table 1:** Characteristics of subjects with COPD, smokers with normal pulmonary function and the unclassified* group

	**UNAFFECTED SMOKERS** **(ppFEV_1 _≥ 80 and FEV_1_/FVC ratio(%) ≥ 70, n = 311)**	**COPD** **(ppFEV_1 _< 75 and FEV_1_/FVC (%) < 70, n = 287)**	Unclassified* n = 281	
	**Mean, SD**	**Mean, SD**	**Mean, SD**	**p Value^†^**

pack years	45.9, 24.7	58.6, 31.1	55.3, 28.3	<0.001
Age	64.4, 10.0	67.3, 8.0	66.1, 9.4	<0.001
FEV_1 _(L/sec)	3.1, 0.5	1.7, 0.6	2.5, 0.53	<0.001
ppFEV_1_	94.7, 10.1	53.5, 14.0	75.9, 12.9	<0.001
FEV_1_/FVC (%)	78.3, 6.0	55.3, 10.8	52.4, 17.7	<0.001
ppFEF_25–75_	85.5, 23.6	26.6, 13.4	71.1, 7.73	<0.001
% male	95.8	97.2	98.2	0.22

*Of 880 Caucasian who smoked ≥ 20 pack years and were older than 50 years, 281 fell into the group excluded from categorical analyses (FEV1/FVC ratio ≥ 70% and ppFEV_1 _< 80% or FEV1/FVC ratio < 70% and ppFEV_1 _≥ 75%, unclassified). The analysis shows significant differences in age, and pack years smoked. ppFEV_1_, FEV1/FVC ratio, and ppFEF_25–75 _were different due to selection criteria.

† Chi-square for sex and ANOVA for the rest of the variables

**Figure 1:**
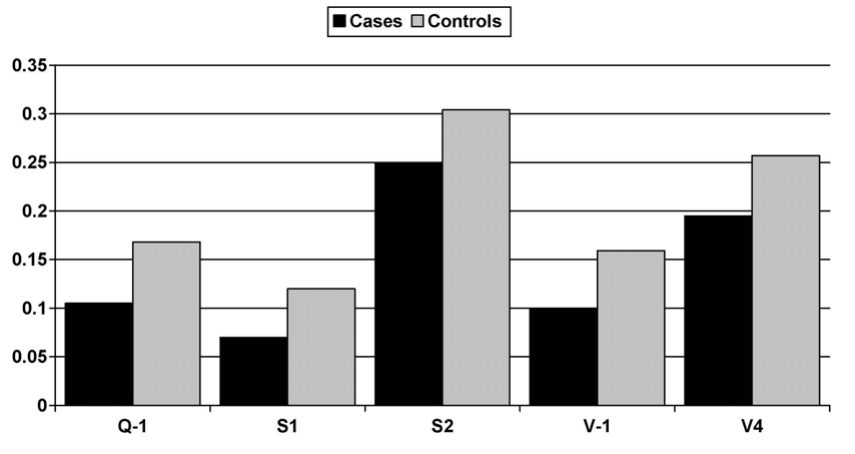
Minor allele frequency of SNPs in ADAM33 that were statistically significantly* different between COPD† cases and controls. *p value < 0.05. SNPs S2 and V4 were not significant after banferroni correction. †COPD: Chronic Obstructive Pulmonary Disease; defined by defined by an FEV1/FVC ratio < 70% and ppFEV_1 _< 75% (n = 287). Control group were smokers with an FEV1/FVC ratio ≥ 70% and ppFEV_1 _≥ 80% (n = 311).

All genotype frequencies were consistent with Hardy-Weinberg equilibrium (p value > 0.05). We observed significant evidence (p value < 0.05) for association between 5 SNPs in ADAM33 (Q-1, rs6127096, p < 0.0028; S1, rs391839, p < 0.0025; S2, rs528557, p < 0.0326; V-1, rs543749, p < 0.0011 and V-4, rs2787094, p < 0.0068, Table 2) and the composite variable for COPD (FEV1/FVC ratio < 70% and ppFEV_1 _< 75%, Figure 1). For these five SNPs, subjects homozygous for the common major allele were more frequent in the COPD group (Figure 1). Inclusion of potential confounders, age, sex, pack-years smoked, smoking status (current versus ex-smoker) and ILO score did not affect the results. After Bonferroni correction, only SNPs S1 and V-1 were significant (p value < 0.0026, based on 19 tests).

**Table 2:** Associations* between SNPs in ADAM33 gene and COPD

		**COPD**		**Controls**		
				
**SNP**	**Genotype**	**N**	**%**	**N**	**%**	p value*
rs2853211	GG	16	5	20	6	
(IVS1_729)	GC	90	31	105	34	0.4083
(AB+)	CC	180	63	185	60	
						
rs4987245	AA	3	1	1	0	
(IVS1_379)	AG	51	18	45	15	0.1522
	GG	231	81	261	85	
						
rs570269	GG	14	05	10	3	
(IVS2_488)	GC	81	28	93	30	0.736
	CC	190	67	205	66	
						
rs487377	AA	15	3	11	5	
(IVS2_1141)	AG	97	29	90	34	0.0826
(BC+1)	GG	174	67	208	61	
						
rs2853210	AA	9	3	13	4	
(IVS2_421)	AG	99	35	121	39	0.1519
	GG	178	62	175	57	
						
rs511898	AA	35	12	44	14	
(IVS6_66)	AG	116	41	136	44	0.2042
(F+1)	GG	134	47	129	42	
						
rs3918395	TT	5	2	4	1	
(IVS13_35)	GT	67	24	74	24	0.8832
(M+1)	GG	212	75	231	75	
						
rs612709	TT	4	1	5	2	
(IVS16_21)	CT	54	19	94	30	0.0028
(Q-1)	CC	226	80	210	48	
						
rs3918396	AA	2	1	3	1	
(Ile710val)	AG	35	12	68	22	0.0025†
(S1)	GG	247	87	237	77	
						
rs528557	CC	17	6	27	9	
(Gly717Gly)	CG	107	38	133	43	0.0326
(S2)	GG	160	56	148	48	
						
rs2853209	TT	60	21	77	25	
(IVS19_181)	AT	144	50	162	52	0.0921
	AA	81	28	70	23	
						
rs598418	CC	38	13	31	10	
(IVS19_384)	CT	147	52	153	49	0.1062
	TT	100	35	125	40	
						
rs44707	CC	49	17	42	13	
(IVS19_427)	CA	143	50	151	49	0.1554
(ST+4)	AA	94	33	115	37	
						
rs574174	AA	7	2	12	4	
(IVS19_959)	GA	79	28	102	33	0.0731
(ST+7)	GG	199	70	196	63	
						
rs2280091	CC	6	2	5	1	
(Met738Thr)	CT	69	24	77	25	0.8823
(T1)	TT	223	74	211	73	
						
rs678881	GG	19	7	12	4	
(IVS21_143)	CG	116	41	115	37	0.0592
	CC	149	52	182	59	
						
rs2787094	GG	10	4	18	6	
(3UTR_449)	CG	90	31	123	40	0.0068
(V4)	CC	186	65	168	54	
						
rs543749	AA	2	1	4	1	
(IVS21_32)	AC	52	18	90	29	0.0011†
(V-1)	CC	233	81	214	69	
						
rs677044	CC	16	6	17	5	
(3UTR_179)	TC	99	35	98	32	0.4908
	TT	170	60	195	63	

*Chi-square test for trend, assuming an additive model (that the risk of the heterozygote genotype is between the risks of the major and the minor homozygote genotypes).

†Significant after Bonferroni correction.

The following SNPs: rs11905870, rs621394, rs17513895, rs615436, rs3918392 and rs3918400 had a minor allele frequency < 0.05 in this population. COPD was associated with the Q-1, S1, S2, V-1 and V4 genotypes. COPD was defined by an FEV1/FVC ratio < 70% and ppFEV_1 _< 75% (n = 287). Control group were smokers with an FEV1/FVC ratio ≥ 70% and ppFEV_1 _≥ 80% (n = 311).

For Q-1, S1 and V-1, quantitative measurements, ppFEV_1_, FEV1/FVC ratio and ppFEF_25–7_, were significantly different between the common homozygous genotypes and other genotypes (dominant genetic model) (Table 3). S2 was associated only with FEV1/FVC ratio and V-4 was not associated with any of the quantitative measurements of pulmonary function (Table 3). Evaluation of all subjects, including the 281 subjects who were not characterized as cases and controls (FEV1/FVC ratio ≥ 70% and ppFEV_1 _< 80% or FEV1/FVC ratio < 70% and ppFEV_1 _≥ 75%), revealed similar results for quantitative traits (Table 3, bold face p values). A subset of this population (n = 453) had information on percent predicted residual volume (ppRV). In these subjects the associations between ppRV and these SNPs showed a trend toward significance only for S1 (mean ppRV = 132.1 for the common genotype, n = 379, and ppRV = 118.4 for the less common genotypes, n = 74, p value < 0.07).

**Table 3:** Estimated* mean pulmonary function measurements for genotypes of SNPs in ADAM33 gene that were associated with COPD.

**SNP**	**Genotype**	**ppFEV1**	p value*	**ppFVC**	p value*	**Ratio**	p value*	**ppFEF25–75**	p value*
									
rs612709	CT+TT	78.62	0.0135	84.49	0.2610	69.88	0.0044	64.52	0.0015
(Q-1)	CC†	73.72	0.0132	83.36	0.3093	66.42	0.0122	55.00	0.0012
									
rs3918396	AG+AA	79.08	0.0256	83.85	0.2883	70.35	0.0068	64.84	0.0079
(S1)	GG†	74.17	0.0143	83.93	0.2710	66.69	0.0112	56.00	0.0019
									
rs528557	CG+CC	75.94	0.2342	84.91	0.8372	68.38	0.0425	59.51	0.1131
(S2)	GG†	74.26	0.1571	83.49	0.6225	66.42	0.1329	55.80	0.1594
									
rs543749	CA+AA	79.08	0.0083	85.20	0.2085	69.96	0.0050	65.17	0.0009
(V-1)	CC†	73.57	0.0057	83.42	0.2211	66.40	0.0116	54.76	0.0004
									
rs2787094	CG+GG	76.50	0.1501	85.14	0.3591	68.32	0.1229	60.54	0.0568
(V4)	CC†	73.92	0.1697	83.61	0.5695	66.63	0.1756	55.32	0.0344

COPD: Chronic Obstructive Pulmonary Disease; defined by an FEV1/FVC ratio < 70% and ppFEV_1 _< 75% (n = 287). Control group were smokers with an FEV1/FVC ratio ≥ 70% and ppFEV_1 _≥ 80% (n = 311).

ppFEV1: percent predicted Forced Expiratory Volume at the First second.

ppFVC: percent predicted Forced Vital Capacity.

Ratio: FEV1/FVC ratio.

ppFEF25–75: Forced Expiratory Flow 25–75%.

* Generalized linear models, adjusted for sex, age and pack-year smoked. Values in bold are pertinent to all subjects (n = 880).

† Major allele homozygous.

**Figure 2:**
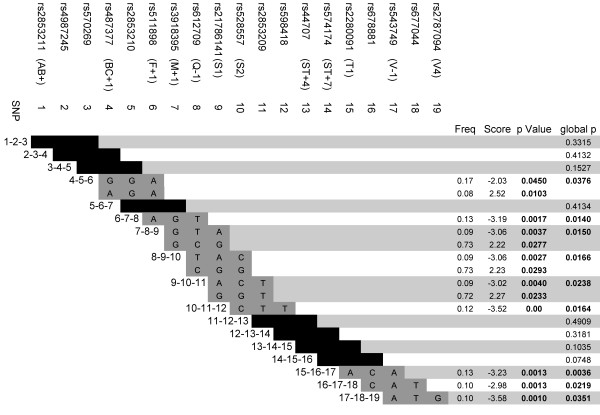
**Haplotype analysis using a sliding window of three SNPs at a time for 19 SNPs with a MAF ≥ 5% in ADAM33 gene, having COPD as the phenotype of interest**.

Haplotype analysis for the 19 SNPs with a MAF > 0.05 was performed using a sliding window to include 3 SNPs at a time. Haplotypes in three regions of the gene were significantly associated with COPD (Figure 2). The second and the third regions included SNPs that showed significance in individual SNP analysis (Q-1-S1-S2 and V-1-V4, respectively). Eight of the thirteen haplotypes were significantly associated with COPD included SNPs Q-1, S1 and S2. SNP S1 was present in six out of these eight SNPs. Linkage disequilibrium between the SNPs measured as D' and r^2 ^are provided in supplemental materials (additional file 2 and additional file 3). In general, the correlation between SNPs was relatively low, but there were high LD measures between SNPs Q-1, S1 and S2 and V-1

## Discussion

In this study we genotyped 880 non-Hispanic whites with a long-term history of cigarette smoking for 25 SNPs in ADAM33. Cases were subjects who met GOLD criteria for stages 2, 3 and 4. The control group for these association studies included chronic cigarette smokers without evidence of airway obstruction. The analysis showed that 5 SNPs (Q-1, S1, S2, V-1 and V4) in ADAM33 were associated with COPD in these smokers. Consistent with these findings, subjects with the rare allele of Q-1, S1, and V-1 had significantly higher values for ppFEV_1_, FEV1/FVC ratio and ppFEF_25–75 _than did subjects with the common allele.

ADAM33, on chromosome 20p13, was identified by positional cloning and was shown to be associated with asthma and bronchial hyper-responsiveness [8]. Since that original publication several studies have replicated the association of ADAM33 with asthma [9,10,12,15,16,24-26]. Howard and coworkers showed an association of ADAM33 with asthma in ethnically diverse populations [9].

Since that report, replication studies in subjects derived from populations in Germany, the United Kingdom, Japan, Australia and the United States have been published [10,12,15]. However, in some studies the association between ADAM33 polymorphisms and asthma susceptibility could not be confirmed [13,14,27].

Previous studies have also demonstrated an association between ADAM33 polymorphisms and measurements of lung function. In a cohort of 200 asthma patients followed over 20 years, Jongepier and coworkers genotyped 8 SNPs in ADAM33 and found that the rare alleles of the SNPs S2, T1 and T2 of ADAM33 were associated with an excess decline in FEV_1_[15]. On a on a population-based birth cohort, Simpson and coworkers reported that carriers of the rare allele of F+1 SNP had reduced lung function at age 3 years. When the recessive model was considered, SNPs F+1, S1, ST+5, and V4 showed association with reduced lung function at age 5 years. Using linkage disequilibrium mapping, they found evidence of a significant causal location between BC+1 and F1 SNPs, at the 5' end of the gene. Four SNPs were associated with lower FEV_1 _(F+1, M+1, T1, and T2). They concluded that polymorphisms in ADAM33 predict impaired early-life lung function.

A relationship between ADAM33 variation and COPD has also been shown. In a Dutch general population including smokers and non-smokers, van Diemen and colleagues genotyped 1390 subject for 8 SNPs in ADAM33. They defined 186 subjects as COPD GOLD stage 2 or greater (FEV1/FVC ratio < 70% and ppFEV1 < 80%). This study showed that individuals homozygous for the minor alleles of SNPs S2 and Q-1 and heterozygous for SNP S1 had an excess annual decline in FEV1 compared to their respective wild type. They also found a significantly greater frequency of minor alleles of SNPs F+1, S1, S2, and T2 in subjects with COPD (n = 186) compared to the entire general population that included non-smokers. Using 111 COPD patients from this population, Gosman et al. suggested association between SNPs ST+5, T1 and T2, and S2 with airway hyper-responsiveness, higher numbers of sputum inflammatory cells and CD8 cells in bronchial biopsies. The Van Diemen study is the only previous study on the association between ADAM33 and COPD. As in Van Diemen's report we saw associations between SNPs Q-1, S1 and S2 and COPD; however with opposite allele. Other differences between that study and the current report are the number of COPD subjects (186 versus 288), the type of control group for COPD (general population vs smokers) and the number of SNPs studied (8 vs 25). Indeed, we believe that the most appropriate control group for studies on COPD should consist of chronic cigarette smokers who are at risk for COPD and yet have normal lung function. To this end, the controls in this report have comparable exposure to tobacco smoke as the affected cases.

The five SNPs that reached statistical significance in our analyses (Q-1, S1, S2, V-1 and V4) were among SNPs that were reported to be significant in the initial report by Van Eerdewegh and coworkers. Furthermore, the allele frequency in both controls and cases are comparable between this report and Van Eerdewegh (cases having COPD and asthma, respectively, Table 4). Frequencies of S2, V-1 and V4 were also comparable to Howard et al [9]. However, the risk alleles in our study were opposite to what were reported by Simpson and van Diemen [16,17]. These five SNPs are confined to two regions in ADAM33 gene (one containing Q-1, S1 and S2 and the other containing V-1 and V4). SNPs Q-1, S1 and S2 are in a block and SNP V-1, although more than 2 kb apart, has high LD measurements (D' = 1 and 0.39 ≤ r^2 ^≤ 0.90) with the SNPs in this block. SNP V4 is neither in a block with its neighboring SNP V1 nor in LD with either of Q-1, S1 or S2. Furthermore, SNP V4 was not associated with any of the lung function measurements. With regard to location and function, SNPs Q-1 and V-1 are in intronic regions, S1 is a non-synonymous and S2 is a synonymous SNP. It is of importance that haplotype analysis showed that S1 was present in 6 out of 13 significant haplotypes. Three of the six haplotypes containing S1 had a frequency of more than 70%, unlike any other SNP. Additionally, S1 was the only SNP whose association with residual volume approached significance (p < 0.07) in a subset of the studied population. While it is possible that Q-1 and V-1 have some effect on mRNA splicing, we hypothesize that S1 accounts for the association with COPD. However, definitive identification of the specific SNP associated with COPD requires functional analysis.

**Table 4:** Comparison of minor allele frequencies between the current study and the original report on ADAM33

	**COPD**		**Van Eerdewegh, All**	
**SNP**	**Controls**	**Cases**	**Controls**	**Cases**

Q-1 (rs612709)	0.168	0.105	0.150	0.088
S1 (rs3918396)	0.120	0.070	0.105	0.054
S2 (rs528557)	0.304	0.250	0.262	0.200
V-1 (rs543749)	0.159	0.100	0.148	0.076
V4 (rs2787094)	0.257	0.195	0.233	0.164

There is some functional data on ADAM33 protein. For example, Foley et al [28]. reported that the ADAM33 mRNA expression was significantly higher in both moderate and severe asthma compared with mild asthma and controls(p < 0.05). Additionally, immunostaining for ADAM33 was increased in the epithelium, submucosal cells, and smooth muscle in severe asthma compared with mild disease and controls and in bronchial bud during airway morphogenesis. ADAM33 is a disintegrin within the metalloproteinase family. Its association with fetal lung morphogenesis and accelerated rate of decline in FEV1 in adults suggests a role in airway remodeling. Hypothesized mechanisms include release or activation of growth factors and facilitation of migration of fibroblasts or inflammatory cells through the matrix. The trend towards association of ADAM33 with RV is consistent with a role for ADAM33 in airway remodeling that will require study with larger numbers to confirm.

Unique strengths of this study were having the proper control subjects, i.e. smokers susceptible to develop COPD, and a thorough SNP panel. A limitation of our study was that we did not formally test for population stratification.

In summary, we evaluated a well characterized group of cases and controls who were long term tobacco smokers and comprehensively genotyped them for ADAM33 variation. Five polymorphisms: Q-1, S1, S2, V-1 and V4 in ADAM33 were associated with COPD. When we applied Bonferroni correction, only SNPs S1 and V-1 hold statistical significance. SNPs Q-1, S1 and S2 were within 500 bp and in a haplotype block. SNP V-1 was 2 kb apart from this block but revealed high linkage disequilibrium measurements with this block. These four SNPs (Q-1, S1, S2 and V-1) were also associated with lung function measurements. SNP V4 was neither linked to the other four SNPs nor was it associated with lung function. Based on these data and the fact that S1 is a non-synonymous SNP (Isoleucine → Valine), studies to assess the functional significance of this amino acid change in the ADAM33 protein and other functional assays are necessary to understand the biologic basis for the association of ADAM33 variation and obstructive pulmonary diseases.

## Competing interests

The authors declare that they have no competing interests.

## Authors' contributions

JO and DAS established the population. ERB, DAM and JO planned the current study. AS and DAM designed and conducted the statistical analyses. AS compiled the results. GAH and SLZ performed genotyping. All authors contributed in writing the manuscript and approved the final version.

## Supplementary Material

Additional File 1 **Asbestos screening.** The questionnaire that was used to obtain information on study subjects. Click here for file

Additional File 2 **D-prime. **The figure represents Linkage disequilibrium (D') between ADAM33 SNPs. Click here for file

Additional File 3 **R-prime.** The figure represents Linkage disequilibrium (r^2^) between ADAM33 SNPs. Click here for file
