# Supplementary material for: Adam33 polymorphisms are associated with COPD and lung function in long-term tobacco smokers
Source: Respir Res. 2009 Mar 12;10(1):21. doi: 10.1186/1465-9921-10-21 (PMC2664793; doi:10.1186/1465-9921-10-21)
Supplement: Additional File 1 — Asbestos screening. The questionnaire that was used to obtain information on study subjects. [file 1465-9921-10-21-S1.doc]

***Asbestos Screening***

Wake Forest University Health Sciences

**Pulmonary Occupational Medicine Questionnaire**

*Revised format: 7/31/2002*

#####

**1. Name_**___________________________________ **12**. **Salary range now or at retirement.**

Less than $10,000 __ $10,000-$20,000 __

**2. Social Security #___________________________** $20,001-$30,000 __ $30,001-$40,000 __

$40,001-$50,000 __ $50,001-$60,000 __

**3. Date of Birth ____________________________** More than $60,000 ___

**4. Age______**

**13. Highest grade completed in school? ___**

**5. Sex _____**

**6. *COMPLETE* Address**_____________________ **14. Personal**

**___________________________ Physician____________________________**

***zipcode* ___________________________**

1. **Telephone number**  daytime (____)_____________ **15. Physician's *complete*** _________________

evening ( )_____________ **address, with _______________________**  **zipcode ____________________________**

1. **Height___________**
2. **Weight___________ 16. Physican's telephone number ( ) ______________**

**10. Marital Status** a. Single _____

b. Married _____

c. Separated/Divorced ____ ***Please, sign here if you wish a copy of this***

d. Widowed ____ ***report sent to your physician.***


**11. Race** a. Asian ____ d. Native American ___ ___________________________________

b. Black ____ e. White ___

c. Hispanic ___ f. Other ___

| **FOR CLINICAL ENTRIES ONLY**  **Code # of sample ­­­­­­­­_________ Today's date_______________**    **Diagnosis Date of diagnosis**  **Mesothelioma ____ ___________________**  **Lung cancer ____ ___________________**  **Asbestosis ____ ___________________**  **Pleural Plaque ____ ___________________**  **Examining physician__________________** |
| --- |

**HEALTH HISTORY**

**17. Have you ever been hospitalized?** Yes___ No___

If you have ever been hospitalized, please answer the following questions.

1. For what condition were you hospitalized?_______________________________________________
2. For how long were you hospitalized? ___________________________________________________
3. When were you hospitalized? _________________________________________________________
4. For what other condition were you hospitalized?__________________________________________
5. For how long were you hospitalized on this occasion? _____________________________________

F. When were you hospitalized? _________________________________________________________

G. For what other condition were you hospitalized?__________________________________________

1. For how long were you hospitalized for this condition? ____________________________________
2. When were you hospitalized? _________________________________________________________

**18. List ALL medications which you are currently taking**. _______________________________________

**________________________________________________________________________________________________________________________________________________________________________________________________________________________________________________________________________________________________________________________________________________________________________**

**__________**

1. **If known, WHEN and where did you have your most recent chest x-ray?**

**____________________________________________________________________**________________

**20. If disabled from walking by any condition other than heart or lung disease, please** **describe and**

**proceed to question 24.__________________________________________________________________**

**21. Are you short of breath when hurrying on level ground or walking up a slight hill?** Yes__ No___

1. **If you are short of breath when hurrying on level ground or walking up a slight hill,**

A. …how long has this been present? _____________________

B. …is this worse now than when it started? Yes__ No___

**22. Whether or not you are short of breath as described above, please, answer these questions.**

A. Do you walk slower than people your age on the level because of breathlessness? Yes___ No___ B. Do you stop for breath when walking at your own pace on level ground? Yes___ No___

C. Are you breathless while dressing or climbing a flight of stairs? Yes___ No___

1. **If short of breath when climbing stairs, how many steps cause you to be short of breath? __________**

**24. Does eating cause you to become short of breath?** Yes___ No___

**25. Do you become short of breath while trying to sleep?** Yes___ No___

**26. Do you ever have spells of chest pain?** Yes___ No___

**27. If you do have spells of chest pain, choose ONE answer for each of the following questions.**

1. You have spells of chest pain:

once a day____ once a week_____ once a month_____ once a year______

1. Your chest pain usually lasts for:

seconds ____ minutes ______ hours _____ days _____

1. How long have you been having spells of chest pain?

Less than a week? ___ More than a week, but less than a month? ____

More than a month, but less than a year? ___ More than a year?___

1. What type of chest pain do you experience? Dull____ Sharp____ Dull AND Sharp ____
2. Can you identify anything that provokes the chest pain such as:

Exercise___ rest___ movement___ other_______________________________

1. Can you identify anything that lessens the chest pain such as:

Exercise___ rest___ movement___ other_______________________________

**28. Do you wheeze (make a musical sound) in your chest when you breathe?** Yes___ No___

1. A. If you wheeze, does this happen:
2. daily? _____ weekly? _____ monthly? _____
4. If you wheeze, how long has this been occurring?

Less than a year? ______ More than a year? _______

**29. Do you cough on most days?** Yes___ No___

A. If you cough on most days, is it dry and hacking? Yes___ No___

B. If you cough on most days, do you usually cough up phlegm? Yes___ No___

C. If you cough on most days, do you usually cough up blood? Yes___ No___

D. For how many years have you been coughing on most days? __________________

**30. Have YOU ever had any of the following conditions?**

Yes No not applicable **If yes, please state when it was diagnosed.**

**allergies ___ ___ _________ ____________________________________**

**asthma** ___ ___ _________ ____________________________________

**bronchitis ___ ___ _________ ____________________________________**

**colon cancer** ___ ___ _________ ____________________________________

**emphysema** ___ ___ _________ ____________________________________

**respiratory tract infection** ___ ___ _________ ____________________________________

**intestinal cancer** ___ ___ _________ ____________________________________

**lung cancer** ___ ___ _________ ____________________________________

**prostate cancer** ___ ___ _________ ____________________________________

**stomach cancer** ___ ___ _________ ____________________________________

**throat cancer** ___ ___ _________ ____________________________________

**rib fractures** ___ ___ _________ ____________________________________

**pneumonia** ___ ___ _________ ____________________________________

**tuberculosis** ___ ___ _________ ____________________________________

**FAMILY HISTORY**

**31. Was either of your biological parents ever told** **that he or she had any of the following conditions?**

*FATHER* *MOTHER*

Yes No Do not know Yes No Do not know

asthma ___ ___ ___ ___ ___ ___

colon cancer ___ ___ ___ ___ ___ ___

emphysema ___ ___ ___ ___ ___ ___

lung cancer ___ ___ ___ ___ ___ ___

other chest ailments ___ ___ ___ ___ ___ ___

prostate cancer ___ ___ ___ ___ ___ ___

stomach cancer ___ ___ ___ ___ ___ ___

intestinal cancer ___ ___ ___ ___ ___ ___

throat cancer ___ ___ ___ ___ ___ ___

If either parent was treated for an illness not listed above, what was the illness? __________________________

Please specify: Father's date of birth___________ Mother's date of birth__________

If father is living, enter his age ___ If mother is living, enter her age___

Age at death if deceased____ Age at death if deceased___

Cause of death if deceased_____ Cause of death if deceased______

Do not know cause of death____ Do not know cause of death____

**32. How many brothers and/or sisters have you ever had? Brothers _______ Sisters _______**

**33. How many daughters and/or sons you ever had? Daughters ______ Sons _______**

1. **Has any member of your family (for example: spouse, children, brother, sister, aunt, uncle)** **ever been diagnosed with any of the following conditions?**

**Asthma:**

Yes No not applicable **If yes,** please state when it was diagnosed**.**

spouse ___ ___ _________ __________________________________________

daughter ___ ___ _________ __________________________________________

son ___ ­­­___ _________ __________________________________________

brother ___ ___ _________ __________________________________________

sister ___ ___ _________ __________________________________________

other ___ ___ _________ __________________________________________

**34. *(continued)* Has any member of your family ever been diagnosed with the following conditions?**

**Colon cancer:**

Yes No not applicable **If yes,** please state when it was diagnosed**.**

spouse ___ ___ _________ __________________________________________

daughter ___ ___ _________ __________________________________________

son ___ ___ _________ __________________________________________

brother ___ ___ _________ __________________________________________

sister ___ ___ _________ __________________________________________

other ___ ___ _________ __________________________________________

**Emphysema:**

Yes No not applicable **If yes,** please state when it was diagnosed**.**

spouse ___ ___ _________ __________________________________________

daughter ___ ___ _________ __________________________________________

son ___ ___ _________ __________________________________________

brother ___ ___ _________ __________________________________________

sister ___ ___ _________ __________________________________________

other ___ ___ _________ __________________________________________

**Intestinal cancer:**

Yes No not applicable **If yes,** please state when it was diagnosed**.**

spouse ___ ___ __________ __________________________________________

daughter ___ ___ __________ __________________________________________

son ___ ___ __________ __________________________________________

brother ___ ___ __________ __________________________________________

sister ___ ___ __________ __________________________________________

other ___ ___ __________ __________________________________________

**Lung cancer:**

Yes No not applicable **If yes,** please state when it was diagnosed**.**

spouse ___ ___ __________ __________________________________________

daughter ___ ___ __________ __________________________________________

son ___ ___ __________ __________________________________________

brother ___ ___ __________ __________________________________________

sister ___ ___ __________ __________________________________________

other ___ ___ __________ __________________________________________

**Prostate cancer:**

Yes No not applicable **If yes,** please state when it was diagnosed**.**

son ___ ___ __________ _________________________________________

husband ___ ___ __________ _________________________________________

brother ___ ___ __________ _________________________________________

other ___ ___ __________ _________________________________________

**34. *(continued)* Has any member of your family ever been diagnosed with the following conditions?**

**Stomach cancer:**

Yes No not applicable **If yes, please state when it was diagnosed.**

spouse ___ ___ __________ __________________________________________

daughter ___ ___ __________ __________________________________________

son ___ ___ __________ __________________________________________

brother ___ ___ __________ __________________________________________

sister ___ ___ __________ __________________________________________

other ___ ___ __________ _________________________________________

**Throat cancer**

Yes No not applicable **If yes, please state when it was diagnosed.**

spouse ___ ___ _________ _________________________________________

daughter ___ ___ _________ _________________________________________

son ___ ___ _________ _________________________________________

brother ___ ___ _________ _________________________________________

sister ___ ___ _________ _________________________________________

other ___ ___ _________ _________________________________________

#### SMOKING HISTORY

**35. Have you ever smoked CIGARETTES?** Yes___ No___

IF YES, please, answer the following questions.

A. How old were you when you first **started** regular cigarette smoking? Age ______

B. What is the **GREATEST** number of cigarettes you EVER smoked **PER DAY**? __________

C. Have you stopped smoking cigarettes completely (as of 1 month ago)? Yes___ No___

D. If you have stopped smoking completely, how old were you when you **quit**? Age ______

**36. Have you ever smoked a PIPE regularly?** Yes___ No___

IF YES, please, answer the following questions.

A. How old were you when you started to smoke a pipe regularly? Age ____

B. What is the **GREATEST** number of pipe fulls you EVER smoked **PER WEEK**? ______________

C. Have you stopped smoking a pipe completely (as of 1 month ago)? Yes__ No___

1. If you have stopped smoking a pipe completely, how old were you when you stopped? Age ____

**37. Have you ever smoked CIGARS regularly?** Yes__ ­ No__

IF YES, please, answer the following questions.

A. How old were you when you started smoking cigars regularly? Age ___

1. What is the **GREATEST** number of cigars you EVER smoked **PER WEEK**? _________________
2. Have you stopped smoking cigars completely (as of 1 month ago)? Yes__ No__

D. If you have stopped smoking cigars completely, how old were you when you stopped? Age ____

**38. Have you ever used smokeless tobacco (SNUFF/CHEWING TOBACCO)?** Yes__ No__

IF YES, please, answer the following questions.

A. How old were you when you started using smokeless tobacco? Age _____

B. What is the **GREATEST** number of cans of smokeless tobacco you EVER used **PER WEEK**? ____

1. C. Have you stopped using smokeless tobacco completely? Yes___ No___

D. If you have stopped using smokeless tobacco, how old were you when you stopped? Age ____

#### OCCUPATIONAL HISTORY

**39. Have you ever worked:** **40. Have you ever worked as:**

Yes No Yes No

A. a boilermaker ___ ___

A. in a cotton, flax or hemp mill? ___ ___ B. a carpenter? ___ ___

B. in a foundry? ___ ___ C. a chemical worker? ___ ___

C. in a glass works? ___ ___ D. an electrician? ___ ___

D. in a mine? ___ ___ E. an elevator operator? ___ ___

E. in a pottery? ___ ___ F. an insulator? ___ ___

F. in a power plant? ___ ___ G. a lather? ___ ___

G. in a quarry? ___ ___ H. a machinist? ___ ___

H. in a refinery? ___ ___ I. a mechanic? ___ ___

I. or with asbestos? ___ ___ J. a millwright? ___ ___

J. or with sheet rock? ___ ___ K. a pipefitter? ___ ___

L. a plasterer? ___ ___

M. a plumber? ___ ___

N. a sander? ___ ___

O. a sheetmetal worker? ___ ___

P. a steelworker? ___ ___

Q. a welder ___ ___

**41. Please list all unions to which you have belonged____________________________________________**

**__________________________________________________________________________________________**

1. **Where or by whom were work clothes laundered?**

**a. at work______ b. self________ c. spouse_______ d. other ________**

***The work history on this page is very important. Please, be as accurate as possible.***

1. **List *in detail*** **the companies and jobs at which you have worked, include any military service. Describe the tasks you performed, how long you worked at each task and whether or not you *used* a respirator during work. Please, start with the first job held (on a farm, grocery, etc.) until the present, even if you were not exposed to asbestos. Please, attach an extra sheet of paper if needed.**

NAME OF TASKS PERFORMED YEARS SPENT Respirator **USED**

EACH COMPANY JOB TITLE ON EACH JOB AT EACH JOB during work

______________________ ____________________ _________________________________ 19 ( ) to 19 ( ) yes___ no___

______________________ ____________________ _________________________________ 19( ) to 19 ( ) yes___ no___

______________________ ____________________ _________________________________ 19( ) to 19 ( ) yes___ no___

______________________ ____________________ _________________________________ 19( ) to 19 ( ) yes___ no___

______________________ ____________________ _________________________________ 19( ) to 19 ( ) yes___ no___

______________________ ____________________ _________________________________ 19( ) to 19 ( ) yes___ no___

______________________ ____________________ _________________________________ 19( ) to 19 ( ) yes___ no___

______________________ ____________________ _________________________________ 19( ) to 19 ( ) yes___ no___

______________________ ____________________ _________________________________ 19( ) to 19 ( ) yes___ no___

______________________ ____________________ _________________________________ 19( ) to 19 ( ) yes___ no___

______________________ ____________________ _________________________________ 19( ) to 19 ( ) yes___ no___

______________________ ____________________ _________________________________ 19( ) to 19 ( ) yes___ no___

**44. Based on your work history, when do you think you were FIRST exposed to asbestos? __________________________________________**

**Signature of person who completed questionnaire:** ____________________________________________________________________________
