# Supplementary figures and images for: Adam33 polymorphisms are associated with COPD and lung function in long-term tobacco smokers
Source: Respir Res. 2009 Mar 12;10(1):21. doi: 10.1186/1465-9921-10-21 (PMC2664793; doi:10.1186/1465-9921-10-21)

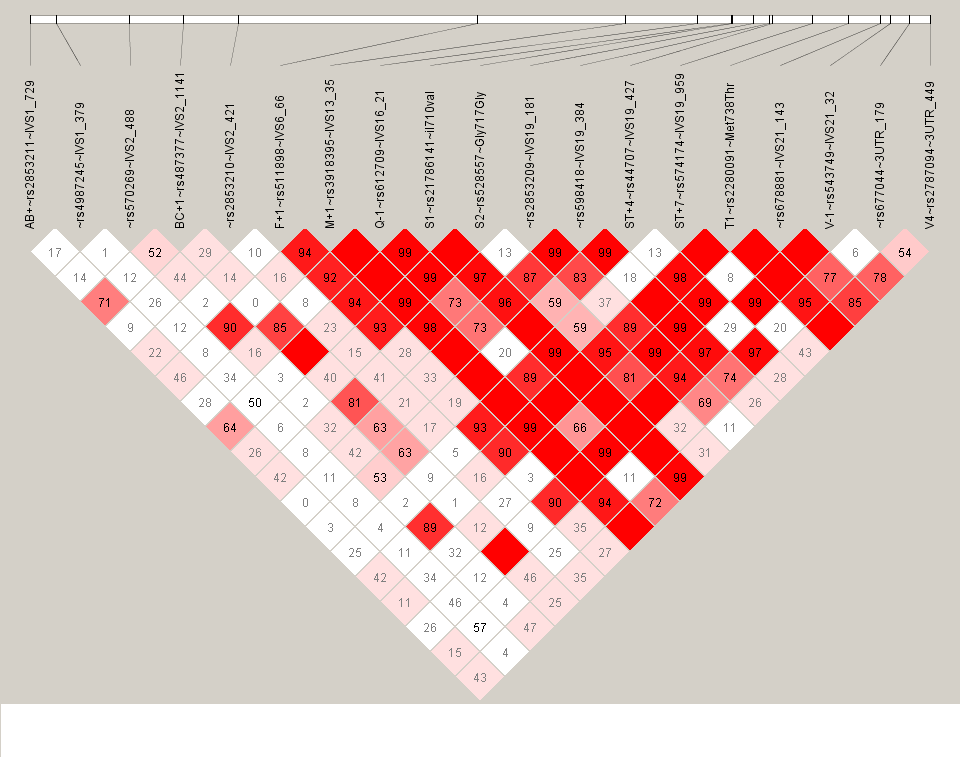

Supplement: Additional File 2 — D-prime. The figure represents Linkage disequilibrium (D') between ADAM33 SNPs. [file 1465-9921-10-21-S2.tiff]

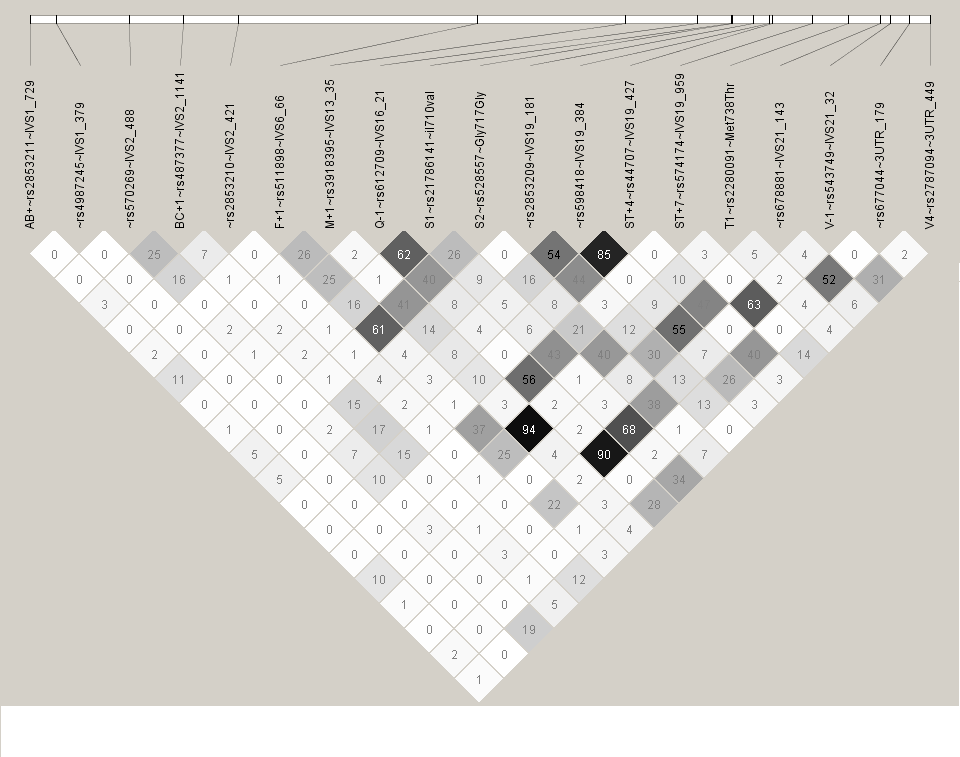

Supplement: Additional File 3 — R-prime. The figure represents Linkage disequilibrium (r2) between ADAM33 SNPs. [file 1465-9921-10-21-S3.tiff]
